# Restore the natural - A review and case series report on reattachment

**DOI:** 10.4317/jced.50948

**Published:** 2014-12-01

**Authors:** Sourabh Jagannath-Torvi, Munniswamy Kala

**Affiliations:** 1 Post Graduate student, Dept of Conservative dentistry and endodontics Govt dental college and research institute, Bangalore; 2 Professor and HOD, Dept of Conservative dentistry and endodontics, Govt dental college and research institute, Bangalore

## Abstract

Reattachment of the original tooth fragment to the fractured tooth helps in maintaining the tooth’s color, wear resistance, morphology and translucency in the restoration. This article describes the reattachment of fractured fragment using a fiber post and dual cure resin cement with a self-etching adhesive. Two young male patients reported with a complicated crown fracture of the right maxillary central incisor due a road traffic accident. The fractured fragments were loosely attached to the palatal gingival tissue, which was then surgically removed and preserved for the reattachment procedure. The fractured tooth segments were successfully reattached following fiber post cementation. Tooth fragment reattachment procedure offers ultraconservative, safe, fast and esthetically pleasing results when the fractured fragment is available due to the improvement of adhesive techniques and restorative materials. Fiber reinforced resins not only allows creation of esthetic restoration but also the preservation and reinforcement of tooth structure. At the 18months follow-up, the resultant appearance was acceptable to the patient.

** Key words:**Reattachment, bonding, complicated crown fracture, fibre post, resin cement.

## Introduction

Tooth injuries constitute an integral part of clinical odontology. Dental trauma within the foreseeable future will probably exceed dental caries and periodontal disease as the most significant threat to dental health among youth and will be accompanied by significant economic consequences. The incidence of complicated crown fractures ranges from 2% to 13% of all dental injuries and the most commonly involved tooth is the maxillary central incisors. Injuries to the maxillary anterior region causes significant disfigurement leading to problems associated with the patient’s aesthetics and appearance as well as function. It is a physical as well as a psychological impact on the patient and in children, it may cause significant concern to the parents. Such aesthetically demanding and critical scenarios require quick and logical clinical actions to rehabilitate the patient.

One of the options for managing coronal tooth fractures, especially when there is no or minimal violation of the biological width, is the reattachment of the dental fragment when it is available (1).

Although composite resins do not have hydroxyapatite crystals, dentinal tubules or enamel rods these newer formulations possess secondary optical properties such as translucency, opacity, opalescence, iridescence, fluorescence and surface gloss. There is, however, no synthetic restorative material that can replicate the aesthetic characteristics or color stability of the natural tooth structure (2)

The first published case of reattaching a fractured incisor fragment was reported in 1964 by Chosack A *et al.* (3) Tennery (4), Starkey (5) and Simonsen (6) were the early workers to report the cases of “tooth fragment reattachment”.

Tooth fragment reattachment technique represents an important step in the science and art of restoring fractured anterior teeth. Fragment bonding usually restores the incisal function and surface anatomy perfectly (7) and is probably less traumatic, simple and low cost method. Additionally it establishes superior esthetics, positive emotional and social response from the patient towards the preservation of natural tooth structure (8).

Anterior tooth fragment have been reattached using composite (9), interlocking pins and light cured resins (10).

This article reports two similar cases of Ellis class 3 fractures (11) in the maxillary central incisors, which were treated by reattachment of the fractured fragments using a fiber post luted by a dual cure composite resin.

## Case report

Two young male patients aged between 22 to 25 years reported to the department of conservative dentistry and endodontics, Government dental college and research institute, Bangalore, Karnataka, India within a span of 12 days with the chief complaint of broken upper front right tooth due to a motorcycle road traffic accident. Both the patients reported within 3 days of the incident. Associated with severe throbbing pain on contact with the lower teeth.

On inspection the right maxillary central incisor in both cases had fractured obliquely about 3-4 mm below the CEJ on the labial aspect and 2 mm above the level of CEJ in the palatal aspect compromising the pulp. The fractured fragment was attached to the palatal gingival tissue.

There were no other injuries associated with the soft tissues or alveolar bone on clinical and radiographic examination.

The patients were presented with the following treatment options after the final diagnosis

1. Root canal treatment followed by reattachment of the fractured fragment following gingivectomy

2. Root canal treatment followed by post and core and composite buildup

3. Root canal treatment followed by post and core and crown after orthodontic tooth extrusion

4. Extraction

**Figure 1:**
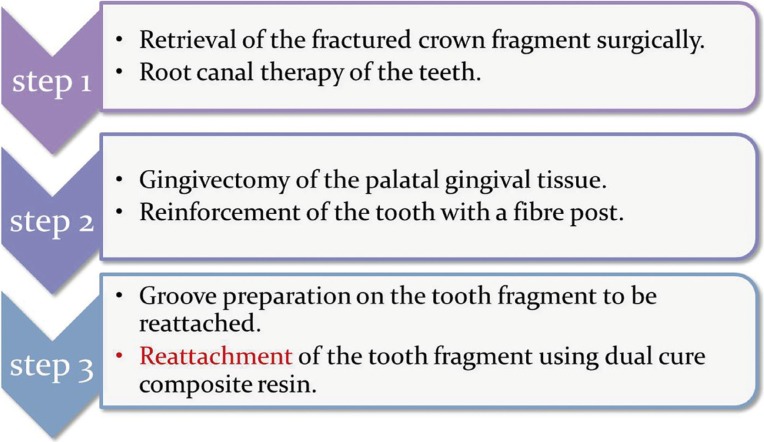
Treatment protocol for fractured fragment reattachment.

Based on the patients need for immediate resuscitation the following treatment protocol was contemplated (Fig. 1)

One important complication in these cases was that the sub gingival extension of the fractured margin on the lingual aspect as mentioned earlier. The gingival aspect of the fractured site revealed a shallow, knife-edge sub gingival fracture margin. Upon probing this area during the clinical examination, it was determined that the biological width was only minimally invaded and that crown lengthening alone in the palatal aspect would be sufficient for access and isolation during the reattachment procedure.

Root canal therapy was completed in a single sitting using AH plus sealer and Gutta percha cones. Crown lengthening was done using electro cautery and 2 mm of palatal tissue was excised. Immediate post space preparation was completed in both cases leaving behind 5 mm of gutta percha in the apical region.

A prefabricated fiber post (Parapost-Fiber lux, Coltene Whaledent) was selected. A retentive groove was prepared in the fractured crown fragments in both cases using a large round bur to act as a retentive area and to receive the post. The alignment of the coronal fragment was assessed with the post in position. The root canal was then etched using 37% phosphoric acid for 15 seconds and thoroughly rinsed off. Bonding agent (ADPER SINGLE BOND2, 3M ESPE) was then applied to the root canal walls and light-cured for 15 seconds. Bonding agent was also applied to the light transmitting post. Dual cure resin (Rely-X, 3M) was placed in the canal and the fiber post was placed up to the proper length. The inner surface of the coronal fragment was similarly etched and bonded to the tooth with dual cure resin composite. When the original position had been reestablished, excess resin was removed and the area was light cured for40 seconds on each surface, making sure that no displacement of the fragment occurred before adhesive/resin polymerization was complete. The margins were properly finished with diamond burs and polished with a series of Sof-Lex disks (3M ESPE) and diamond polishing paste. The occlusion was carefully checked and adjusted. Instructions were given as to avoid heavy forces on these teeth to both patients and to follow regular oral hygiene practices.

**Figure 2:**
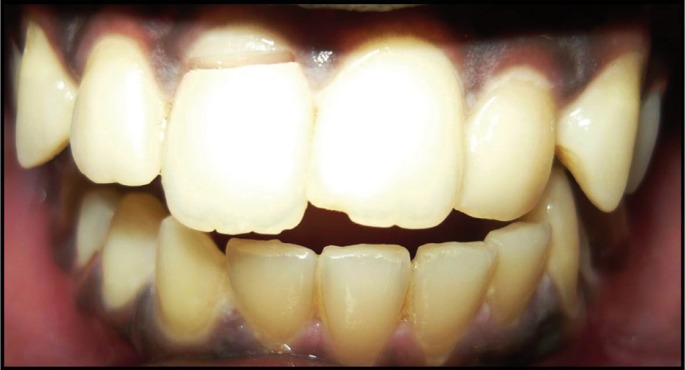
Preoperative Case 1.

**Figure 3:**
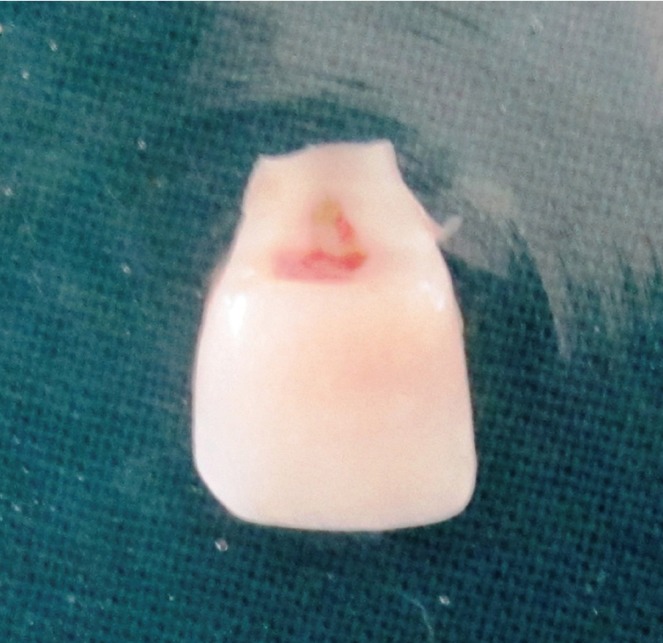
Retrieved Fractured Fragment Case 1.

**Figure 4:**
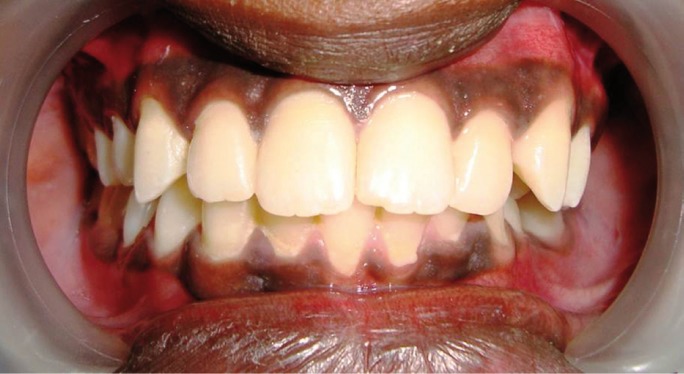
Post operative Case 1.

**Figure 5:**
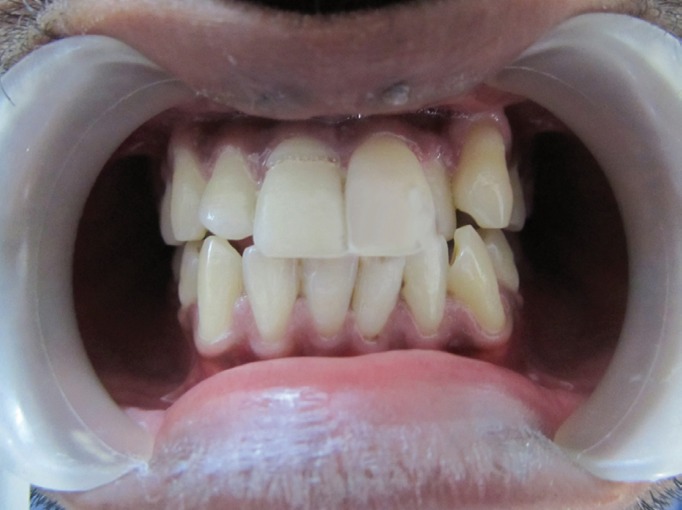
Preoperative Case 2.

**Figure 6:**
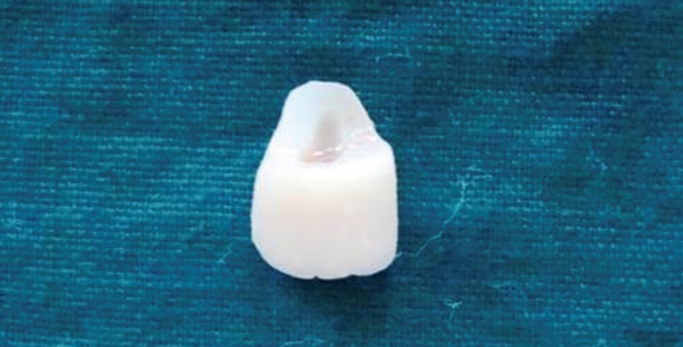
Retrieved Fractured Fragment Case 2.

**Figure 7:**
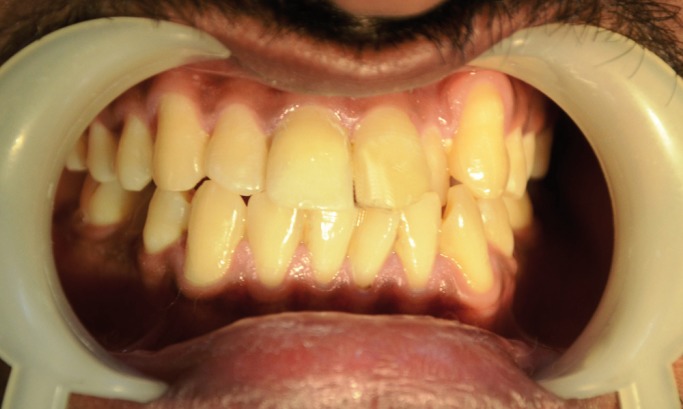
Postoperative Case 2.

The patients returned for 1, 6, 12 and 18-month follow-ups, and restorative treatments remained clinically and aesthetically acceptable for the entire time (Figs. 2-7).

## Discussion

Whenever the fractured fragment is available intact, the reattachment of the fragment has to be the most desired treatment. In recent years due to remarkable advancement of adhesive systems and resin composites has made reattachment procedure no longer a provisional restoration. Fabrication of a mouth guard and patient education about treatment limitations may enhance clinical success as reattachment failures may occur with new trauma or Parafunctional habits (12).

The composite resin has a favorable subgingival reaction and the formation of junctional epithelium and connective tissue adjacent to subgingival restorative materials in humans (13).

But at the same time considering the proper contour and marginal adaptation of subgingival restoration is of prime importance. Bonding of original fragment permits subgingival healing with long thick functional epithelium. The psychological trauma caused to the individual due to the disfigurement can be managed by this procedure successfully and in a shorter period of time as compared to conventional treatment approaches. In the absence of luxation injuries this procedure can be considered (14).

In the pre adhesive era, fractured teeth needed to be restored either with pin retained inlays or cast restorations that sacrificed the healthy tooth structure and were a challenge for clinicians. The development of adhesive dentistry has opened the floodgates to a wide array of techniques which includes the reattachment procedure where the patient’s own tooth fragment can be considered for reattachment. Along with electrosurgical crown lengthening procedure and adequate isolation reattachment can be a logical and justified approach for treating fractured anterior teeth, where the fractured fragment is either attached or preserved in a suitable medium. Use of a fiber post luted with resin cements increases the retention of the segment and provides a monoblock effect (15). Longevity of a tooth fragment reattachment is not foreseeable, but the real merit of reattachment is the fact that all other restorative options, such as direct adhesive ones, veneers, and crowns will always be open. With advancement in dental bonding technology, it is now possible to achieve excellent results with reattachment of fractured tooth fragments, provided that the biologic factors and selection of materials are logically assessed and managed.

The main challenge to a clinician is to manage the psychological impact as well as the physical injury the patient sustains in an accident. In these two case reports both patients were aged within 22 – 25 years and were extremely conscious about their personal appearance. Fragment reattachment procedure was the best logically justified treatment option for these cases. The treatment procedure was conservative, done in a single appointment, provided absolute aesthetics and both patients were satisfied by the results. Follow up recalls up to 18 months have remained satisfactory to the patients as well as the clinician by using sound judgment, following proven protocols the restorative clinician can integrate the reattachment procedure into his or her practice to provide the contemporary dental patient with a viable treatment alternative.
